# Cluster headache: new targets and options for treatment

**DOI:** 10.12688/f1000research.13380.1

**Published:** 2018-03-20

**Authors:** Patty Doesborg, Joost Haan

**Affiliations:** 1 Department of Neurology, Leiden University Medical Center, Leiden, Netherlands; 2 Department of Neurology, Alrijne Ziekenhuis, Leiderdorp, Netherlands

**Keywords:** Cluster headache, treatment targets, Calcitonin gene-related peptide targeted therapies

## Abstract

Cluster headache is a severe headache disorder with considerable impact on quality of life. The pathophysiology of the disease remains poorly understood. With few specific targets for treatment, current guidelines mainly include off-label treatment with medication. However, new targets for possible treatment options are emerging. Calcitonin gene-related peptide (CGRP)-targeted medication could become the first (cluster) headache-specific treatment option. Other exciting new treatment options include invasive and non-invasive neuromodulation techniques. Here, we provide a short overview of new targets and treatment options that are being investigated for cluster headache.

## Introduction

Cluster headache is one of the primary headache disorders. Patients suffer from attacks of severe unilateral pain that is located in the orbital, retro-orbital, or temporal regions and lasts for 15 to 180 minutes. Attacks can occur in a high frequency varying from one to eight times per day. The disease is named after its remarkable feature of attacks clustering in periods lasting for weeks or months, and remission periods occur in between. In chronic cluster headache, these remission periods are absent ^1^.

The disease affects 1 in 1,000 people, and prevalence is higher among men (ratio of 2.5:1) ^2^. Cluster headache has a substantial impact on quality of life. A study among Danish patients showed considerable restrictions to daily activities and working ability ^3^. Other studies have shown lower scores on the 36-Item Short Form Health Survey (SF-36) in patients with episodic cluster headache during an active bout and in chronic cluster headache compared with healthy controls ^4, 5^.

The mechanism behind cluster headache attacks is poorly understood. Activation of the trigemino-vascular reflex could be an explanation for the autonomic symptoms following the severe pain in the trigeminal region. However, this does not explain the rhythmicity of cluster headache. Involvement of the hypothalamus is suggested as an explanation for this distinct clinical feature, and this possibility is supported by hormone and imaging studies. Indeed, several studies have demonstrated altered functioning of the hypothalamus in cluster headache.

Currently available and recommended treatment consists of both acute and prophylactic treatment options ^6^. Cluster headache attacks can be aborted by acute treatment with triptans. There is little evidence on which triptan is most effective. Proven, effective abortive treatments are sumatriptan or zolmitriptan after subcutaneous or intranasal administration ^7, 8^. Owing to their rapid onset of action, these triptans are very suitable for aborting a cluster headache attack. Other options for acute attack treatment include sumatriptan or lidocaine through intranasal administration, oral zolmitriptan, sphenopalatine ganglion (SPG) stimulation, and octreotide through subcutaneous administration. A history of cardiovascular disease is a formal contraindication for the use of triptans. Years of clinical experience have shown that it could be seen as a reason for caution rather than a strict contraindication. Oxygen (100%, 6–12 L/minute) is a proven alternative and is effective in aborting a considerable number of cluster headache attacks ^6^.

Prophylactic treatment with verapamil and lithium currently has the highest level of evidence for efficacy. Other possible effective preventative treatments are warfarin and melatonin ^6, 9, 10^. In daily practice, medication with a lower level of evidence, such as topiramate, frovatriptan, and gabapentin, is sometimes tried. In recent years, suboccipital steroid injections have proven to be effective as a short-term, preventive treatment option in some patients ^6^.

A subgroup of patients with cluster headache does not respond well to the currently available treatment. This can be due to lack of response to the medication or to side effects of the drugs. The most commonly used preventive treatment, verapamil, can cause electrocardiography abnormalities and severe clinical side effects ^11^. Lithium is also not very well tolerated by many patients. Additional preventative treatments are available, but evidence of effectiveness was never properly shown in clinical trials. As mentioned before, patients with cluster headache have a severe disease burden. This severe disease burden calls for an effective treatment. Therefore, there is a need for new and better treatments. Thus far, recommended guidelines for cluster headache mainly include the above-mentioned off-label drugs, orally or through injections ^6^. In the past couple of years, possible new targets for the treatment of cluster headache have emerged, and we will focus on these below.

## Calcitonin gene-related peptide (CGRP)-targeted therapies

The currently available preventative treatments do not target a specific part of the (presumed) cluster headache pathophysiological mechanisms. For many years, it has been hypothesized that neuropeptides play a role in the pathophysiology of both migraine and cluster headache. Based on this assumption, new medication has been developed targeting one of these peptides: calcitonin gene-related peptide (CGRP). In cluster headache, increased saliva and blood levels of CGRP were found during attacks compared with a baseline value in the same patient and healthy controls, suggesting a possible role for CGRP in cluster headache ^12– 15^.

Clinical studies with oral CGRP antagonists in patients with migraine had to be terminated prematurely because of concerns about hepatotoxicity. However, subsequent trials with anti-CGRP monoclonal antibodies in patients with migraine provided strong data for anti-CGRP treatment as a possible new therapy for migraine ^16^.

Four clinical trials are being conducted at this moment to investigate whether the administration of monoclonal antibodies targeting CGRP is effective in preventing cluster headache attacks. Two drugs are under investigation. Two phase III studies are evaluating the efficacy and safety of the anti-CGRP monoclonal antibody fremanezumab for the prevention of both episodic and chronic cluster headache. Additionally, the efficacy and safety of the monoclonal anti-CGRP antibody galcanezumab are being investigated for use in patients with episodic and chronic cluster headache in two phase III, randomized, double-blind, placebo-controlled trials ^17^.

In summary, the likely role of CGRP in cluster headache and the observed efficacy of the antibodies in migraine justify the expectations that these antagonists will also be effective and targeted preventive treatments for cluster headache in the near future.

## Neuromodulation

Neuromodulation could be another new treatment but without the disadvantage of systemic side effects. Several ways of neuromodulation, both invasive and non-invasive, have been investigated recently.

Based on the findings of imaging studies (mainly positron emission tomography and voxel-based morphometry), hypothalamic deep brain stimulation has been tried in patients with medically intractable cluster headache and, in a total of 64 patients, was shown to be successful in 64% of patients with medically intractable chronic cluster headache ^18^. However, this invasive technique has the risk of several possible adverse events, including fatal intracerebral hemorrhage ^18^. There is an ongoing search for less dangerous but equally effective neuromodulation techniques, of which we will discuss peripheral occipital nerve stimulation (ONS), SPG stimulation, and non-invasive vagal nerve stimulation (nVNS).

### Occipital nerve stimulation

Given the observations of involvement of the ipsilateral posterior inferior hypothalamus in cluster headache attacks and the findings from experimental studies, which show convergence of dural afferents and the greater occipital nerve (GON) in the trigeminocervical pathway, it was thought that stimulation of the latter could influence cluster headache ^19, 20^. This idea was supported by multiple open-label studies showing a positive effect of an occipital injection with a corticosteroid: the GON injection ^21– 25^. In addition, two randomized controlled trials showed a significant reduction in cluster headache attacks in both episodic and chronic cluster headache patients after receiving a GON injection ^26, 27^.

Several open-label studies with invasive ONS have been performed in patients with medically intractable chronic cluster headache ^28– 31^. Burns *et al*. showed improvement in 10 out of 14 patients and a marked or moderate improvement in 6 out of 14 patients ^28^. Fontaine *et al*. showed reduction of more than 50% of attacks in 10 out of 13 patients ^31^. Magis *et al*. showed a significant improvement in 80% of 15 patients ^29^. As these were all open studies and therefore possibly influenced by the placebo effect, our department is assessing the efficacy and safety of ONS in patients with medically intractable chronic cluster headache in a randomized blinded trial comparing low with high stimulation ^32^.

### Vagal nerve stimulation

The gammaCORE, a device for nVNS, has been investigated in several studies in both acute and preventive treatment for cluster headache ^33^. Nesbitt *et al*. suggested a role for nVNS in an open-label, observational cohort study in 19 patients with cluster headache ^34^. A prospective, open-label, randomized trial by Gaul *et al*. compared nVNS added to standard of care (SOC) with SOC alone in 45 patients with chronic cluster headache and 48 controls ^35^. A reduction of 5.9 versus 2.1 attacks per week was found in the patient group versus the controls after a 4-week period receiving nVNS. A group of 30 patients continued nVNS for an extension period and reported a further reduction of weekly attack frequency of two attacks per week. The duration or intensity of attacks was not significantly reduced. No serious adverse events were reported ^35^. No randomized controlled trial has been conducted to confirm the additional preventative effect of nVNS in chronic cluster headache.

A double-blind, randomized, sham-controlled trial to evaluate nVNS as an acute treatment in 133 patients with cluster headache showed no significant difference in response rate after nVNS versus sham. However, in a subgroup analysis of patients with episodic cluster headache, a significantly higher response rate was found (34.2% versus 10.6%, *p* = 0.008) ^36^.

The trials conducted so far have not been able to prove the efficacy of nVNS as an alternative to acute or prophylactic treatment in chronic cluster headache ^35, 36^. However, it has been proposed to include nVNS in the guidelines for the treatment of episodic cluster headache ^37^.

### Sphenopalatine ganglion-targeted therapies

Current acute attack treatment in cluster headache consists of either a subcutaneous injection of sumatriptan by means of an auto-injector or oxygen inhalation. Stimulation of the SPG is a new acute treatment under investigation at present. To apply stimulation to the SPG, a miniature device with leads is placed in the pterygopalatine fossa.

An early indication for the effectiveness of SPG stimulation was found in a study of six patients with refractory chronic cluster headache; a pain score reduction of more than 50% was found in 14 out of 18 attacks, and complete remission of pain was reached in 11 out of 18 attacks ^38^. In a randomized placebo-controlled trial by Schoenen *et al*., 32 patients with chronic cluster headache were instructed to treat attacks with the stimulator for 15 minutes when they experienced an attack with a categorical pain scale score of 2 or more ^39^. There was a total of 566 attacks registered, and pain relief was achieved in significantly more attacks after full stimulation compared with sham stimulation (67.1% versus 7.4%, *p* <0.0001). Although the trial was set up as an acute treatment study, a reduction in mean attack frequency was also observed. In the experimental period, 12 out of 28 patients experienced an attack frequency reduction of more than 50% compared with the baseline attack frequency ^39^. In an open-label follow-up of this study, a persistent acute response (in more than 50% of attacks) was found in 45% of patients after 24 months with a total of 4,340 attacks treated. In 78% of these attacks, SPG stimulation was effective. Besides, a reduction of more than 50% in attack frequency as compared with baseline was shown in 33% of patients ^40^. In a recent open-label study in 97 cluster headache patients receiving SPG stimulation, 43 out of 78 patients (55%) with chronic cluster headache experienced a more than 50% reduction in attack frequency after 12 months of stimulation ^41^.

These results indicate that SPG stimulation could be a preventative treatment as well as an acute attack treatment. In the study by Jürgens *et al*. ^40^, cluster attack frequency was defined as the average weekly attack frequency over a 4-week period at baseline compared with a 4-week period after 24 months. It is known that cluster attack frequency can fluctuate over time in patients with chronic cluster headache. This natural fluctuation could be part of the reduction found in these studies.

At present, a randomized controlled trial is investigating both the safety and the efficacy of SPG stimulation in chronic cluster headache ^17^. The SPG can also be targeted by a block or radiofrequency ablation. Available studies on SPG blocks include one small double-blind, placebo-controlled study in 15 patients with cluster headache that studied pain relief after a block with cocaine or lidocaine compared with a saline solution in nitroglycerine-induced attacks. Pain relief was reported after 31.3 (cocaine) and 37 (lidocaine) versus 59.3 (saline) minutes. One small cohort study showed statistically improved attack intensity and frequency in 15 chronic cluster headache patients up to 18 months after radiofrequency ablation. Additional evidence consists of case series or case reports using different types of blocking agent. Sufficient evidence to show the effectiveness of these treatments is lacking ^42^.

## Conclusions

Here, we have given a short clinical overview of recent developments in the field of cluster headache treatment. Medication targeting CGRP and several neuromodulation techniques are currently being investigated. Promising results from many of the studies conducted up to now give hope for more effective treatments and treatment choices for this very severe pain syndrome in the future.
